# Whole Genome Sequence Analysis of *Brucella* spp. from Human, Livestock, and Wildlife in South Africa

**DOI:** 10.1007/s12275-024-00155-8

**Published:** 2024-07-22

**Authors:** Koketso Desiree Mazwi, Kgaugelo Edward Lekota, Barbara Akofo Glover, Francis Babaman Kolo, Ayesha Hassim, Jenny Rossouw, Annelize Jonker, Justnya Maria Wojno, Giuseppe Profiti, Pier Luigi Martelli, Rita Casadio, Katiuscia Zilli, Anna Janowicz, Francesca Marotta, Giuliano Garofolo, Henriette van Heerden

**Affiliations:** 1 Department of Veterinary Tropical Diseases, Faculty of Veterinary Science, University of Pretoria, Onderstepoort, 0110 South Africa; 2 Unit for Environmental Sciences and Management, North-West University, Potchefstroom, 2531 South Africa; 3 Bologna Biocomputing Group, University of Bologna, 40126 Bologna, Italy; 4 National and OIE Reference Laboratory for Brucellosis, Experimental Zooprophylactic Institute of Abruzzo and Molise Giuseppe Caporale, 64100 Teramo, Italy; 5 National Institute for Communicable Diseases, A Division of the National Health Laboratory Services, Johannesburg, 2192 South Africa; 6 Lancet Laboratories, Microbiology Laboratory, Century City, Cape Town, 7441 South Africa

**Keywords:** Brucellosis, *B. abortus*, *B. melitensis*, Livestock, Human, Wildlife

## Abstract

Brucellosis is an economically important zoonotic disease affecting humans, livestock, and wildlife health globally and especially in Africa. *Brucella abortus* and *B. melitensis* have been isolated from human, livestock (cattle and goat), and wildlife (sable) in South Africa (SA) but with little knowledge of the population genomic structure of this pathogen in SA. As whole genome sequencing can assist to differentiate and trace the origin of outbreaks of *Brucella* spp. strains, the whole genomes of retrospective isolates (n = 19) from previous studies were sequenced. Sequences were analysed using average nucleotide identity (ANI), pangenomics, and whole genome single nucleotide polymorphism (wgSNP) to trace the geographical origin of cases of brucellosis circulating in human, cattle, goats, and sable from different provinces in SA. Pangenomics analysis of *B. melitensis* (n = 69) and *B. abortus* (n = 56) was conducted with 19 strains that included *B. abortus* from cattle (n = 3) and *B. melitensis* from a human (n = 1), cattle (n = 1), goat (n = 1), Rev1 vaccine strain (n = 1), and sable (n = 12). Pangenomics analysis of *B. melitensis* genomes, highlighted shared genes, that include 10 hypothetical proteins and genes that encodes for acetyl-coenzyme A synthetase (*acs*), and acylamidase (*aam*) amongst the sable genomes. The wgSNP analysis confirmed the *B. melitensis* isolated from human was more closely related to the goat from the Western Cape Province from the same outbreak than the *B. melitensis* cattle sample from different cases in the Gauteng Province. The *B. melitensis* sable strains could be distinguished from the African lineage, constituting their own African sub-clade. The sequenced *B. abortus* strains clustered in the C2 lineage that is closely related to the isolates from Mozambique and Zimbabwe. This study identified genetically diverse *Brucella* spp. among various hosts in SA. This study expands the limited known knowledge regarding the presence of *B. melitensis* in livestock and humans in SA, further building a foundation for future research on the distribution of the *Brucella* spp. worldwide and its evolutionary background.

**Supplementary Information:**

The online version contains supplementary material available at 10.1007/s12275-024-00155-8.

## Introduction

The genus *Brucella* is a highly monomorphic genus of pathogenic species affecting a wide range of hosts including humans, livestock, and wildlife in SA. Molecular typing test has impacted the differentiation of *Brucella* homogeneity more than biotyping consisting of 25 biochemical tests to identify *Brucella* species and their biovars and the latter’s inability to differentiate atypical *Brucella* species (Ledwaba et al., 2021). Furthermore, biotyping is often difficult to interpret due to limited standardization of the typing reagents (Whatmore, 2009). Eight distinct *B. abortus* biovars (1–7 and 9) have been documented, whereas *B. suis* has five biovars (1–4 and 5) and *B. melitensis* has three biovars (1–3) (Verger et al., 1985). Additionally, there is no biovar differentiation for other *Brucella* spp. (Matle et al., 2021; Verger et al., 1985). Moreover, the efficacy of biotyping is moderate. Since it includes the manipulation of the live agent, it poses a biosafety and public health risks of laboratory infections to the personnel involved (Whatmore, 2009). Recently, more researchers are utilizing whole genome sequencing (WGS) to explain the relationships between different strains of *Brucella*, either supplementing or replacing traditional molecular typing techniques such as multilocus sequence typing (MLST) and multiple locus variable number of tandem repeats analysis (MLVA). The use of whole genome sequencing to characterize the genomes of *Brucella* spp., allows to determine additional traits like virulence factors, mobile genetic elements while acquiring the highest genetic resolution (Wattam et al., 2014).

To improve strain typing, whole-genome sequencing-based analysis that employs Average Nucleotide Identity (ANI) and whole genome single nucleotide polymorphism (wgSNP) (Abdel-Glil et al., 2022; Uelze et al., 2020) have been employed. The diversity of strains implicated in infections may be revealed by wgSNP analysis of isolates from various geographical locations (Anbazhagan et al., 2023). Pan-genome analysis can be used to determine the genetic diversity of related genomes such as strain tracking, niche specialization and diagnostic marker identification. The fundamental components of the biology behind the main behavioural features were controlled by the genes that make up the core genome (Vernikos et al., 2015). When new *Brucella* genomes are sequenced, pan-genome analysis, as opposed to SNP, can compute, and compare existing genomes to estimate the number of protein-coding genes and aid in the understanding the role of accessory genes such as horizontal gene transfer.

Brucellosis is a controlled disease in SA, focused primarily on mitigating the risk of bovine brucellosis caused mainly by *B. abortus* in cattle through a surveillance and disease control scheme and governed by relevant legislation (R.2483 of 9 Dec 1988 of the Animal Diseases Act 35 of 1984). *Brucella* *abortus* is endemic in cattle in SA occurring in all nine provinces (De Massis et al., 2019). The wgSNP analysis of *B. abortus* strains from cattle in SA with global sequences indicated these SA strains grouped closely to *B. abortus* strains from Mozambique and Zimbabwe as well as Eurasian countries such as Portugal and India. Furthermore, the wgSNP analysis suggested that the introduction of infected animals might be the mode of brucellosis transmission between farms in SA (Ledwaba et al., 2021). *Brucella melitensis* normally associated with small livestock, is not subject to routine surveillance, and testing only becomes compulsory for exports or during outbreaks. *Brucella melitensis* has been sporadically reported in sheep and goats in 1965 (Van Drimmelen, 1965), 1989 (Ribeiro et al., 1990), and 1994 (Reichel et al., 1996). *Brucella melitensis* outbreaks from 2014–2016 occurred in humans linked to sheep and goats (Wojno et al., 2016) as well as in sables with outbreaks on the same farm from 2004 to 2015 followed by various infected sable being reported from 2013 to 2018 (Glover et al., 2020). The latter study reported persistent *B. melitensis* infection in sables (Glover et al., 2020). The investigation did not identify local livestock as a source of ongoing infection in sable but rather that the persistent infection is consistent with the disease circulating within herds (ranched wildlife) and is transmitted through the keeping and trading of animals. *Brucella melitensis* was reported for the first time in cattle by Kolo et al., (2019) implying that mixed farming practises results in spread between cattle, sheep and goats.

The *Brucella* spp. strains that were found to be circulating in goats, sable antelope, human samples from South African provinces, and seemingly healthy animals from abattoirs were whole genome sequenced in this study. Using publicly accessible genomes from GenBank, particularly the African genomes in the database, comparative genomics was employed in the current study to determine the taxonomic classification of the *Brucella* spp. Using genome indices like ANI, pangenomics, and wgSNP, the study examined the South African sequenced *Brucella* spp. strains to determine the genetic diversity and to trace the geographical origin of cases of brucellosis.

## Materials and Methods

### Study Area and Sample Collection

*Brucella* isolates and/or DNA characterised and sequenced in this study were recovered from human (blood) (n = 1) (Wojno et al., 2016), abattoir cattle (tissues) (n = 3) (Kolo et al., 2019), farmer’s cattle (tissues) (n = 1) (Ledwaba et al., 2021), farmer’s goat (tissues) (n = 1), sable antelope (*Hippotragus niger*) (tissues, hygroma fluid, joint fluid, skin lesion, serum) (n = 12) (Glover et al., 2020) and *B. melitensis* Rev1 vaccine strain used in SA (Table 1). These were collected from selected provinces of SA including Gauteng, Limpopo, North West, Eastern Cape, and Western Cape. Cattle tissue samples were collected from apparently healthy animals between the years 2016 to 2022 in Gauteng Province (Kolo et al., 2019). The human case of brucellosis was a 27 year old male from Western Cape Province who appeared unwell and on examination he presented with vomiting, diarrhoea and fever (Wojno et al., 2016). After a thorough medical history, it was discovered that the patient had been feeding his dog with abattoir waste from sheep, goats, and cattle dumped at an accessible municipal waste facility (Wojno et al., 2016). The goat sample sequenced in this study was collected from animals that tested brucellosis seropositive during the investigation of the animal source of the human case in the Western Cape Province (Wojno et al., 2016). The sable samples were collected over a 12 year-period (Glover et al., 2020).

**Table 1:** *Brucella* sequenced isolates that were used in this study isolated from various sources

Sample name	Sample ID	Host	Sample source	Province of origin	Bio-typing species identification
Sample 20_S4	FS20 S4	Cattle	Tissue	Gauteng abattoir	*B. abortus*
Sample 48_S5	FS48 S5	Cattle	Tissue	Gauteng abattoir	*B. abortus*
Sample 1.9	1.9	Cattle	Tissue	Gauteng	*B. abortus*
Bmel_S8	Bmel S8	Cattle	Tissue	Gauteng abattoir	*B. melitensis*
Sample 1.10	25010 1 10	Sable	Tissue	Gauteng	*B. melitensis*
Sample 1.15	25010 1 15	Sable	Tissue	Gauteng	*B. melitensis*
B0214	BS0214	Sable	Tissue	Eastern Cape	*B. melitensis*
B0160	BS0160	Sable	Tissue	Eastern Cape	*B. melitensis*
B0487	BS0487	Sable	Joint fluid	Eastern Cape	*B. melitensis*
BS1SKIN	BS1SKIN	Sable	Skin lesion	Limpopo	*B. melitensis*
Sample 8_S8	NIS8	Human	Blood	Western Cape	*B. melitensis*
BS0018	BS0018	Goat	Tissue	Western Cape	*B. melitensis*
2017 TE 25008 1.8	BS2271	Sable	Tissue	Gauteng	*B. melitensis*
2017 TE 25008 1.7	BS2770	Sable	Tissue	North West	*B. melitensis*
2017 TE 25008 1.9	BS04HY	Sable	Hygroma fluid	Eastern Cape	*B. melitensis*
B04 Serum	BS04 Serum	Sable	Serum	Limpopo	*B. melitensis*
BS06 Serum	BS06 Serum	Sable	Serum	Limpopo	*B. melitensis*
Sample 1.14	25010 1 14	Sable	Sable	Gauteng	*B. melitensis*
Sample 1.13	25010 1 13	Unknown	Rev1 Vaccine	n/a	*B. melitensis*

NB, n/a not applicable

Isolates for sequencing were selected based on their representation of diverse host origins (human, livestock, and wildlife) and geographical spread across SA to ensure a comprehensive analysis of *Brucella* spp. diversity. Priority was given to retrospective isolates from previous studies with confirmed *Brucella* infections, aiming to enrich the study's genomic dataset with genetically varied strains. This selection criterion was intended to maximize the potential for uncovering novel insights into the population structure, transmission dynamics, and evolutionary history of *Brucella* spp. within the region.

### Molecular Identification of Isolates

Initially, the genomic DNA from bacterial colonies were verified using *Brucella* genus and species-specific conventional PCR (cPCR) based on the targeted sequences 16S − 23S ribosomal DNA interspacer region (ITS) and IS711 regions using AMOS-PCR. The genus specific ITS PCR produced a PCR product of 214 bp using the protocol previously developed by Keid et al., (2007). With the multiplex AMOS-PCR assay, *B. abortus* bv 1, 2 and 4, *B. melitensis* bv 1, 2 and 3, *B. ovis*, and *B. suis* bv 1 were identified and differentiated as previously reported (Bricker & Halling, 1994). The four species-specific forward primers were used at a final concentration of 0.1 μM with 0.2 μM reverse primer IS711. PCR cycling condition consisted of an initial denaturation at 95 °C for 5 min followed by 35 cycles of 95 °C for 1 min, 55.5 °C for 2 min, 72 °C for 2 min and a final extension step at 72 °C for 10 min. The PCR products were analysed using 2% agarose gel electrophoresis using *B. abortus* bv 1 (REF 544 / BCCN R4), *B. abortus* S19 (Onderstepoort Biological Products) and *B. melitensis* Rev1 (Onderstepoort Biological Products) as positive controls.

### Whole-Genome Sequencing and Biotyping

Following the preceding description, genomic DNA of the *Brucella* spp. isolates (n = 19) were submitted for whole genome sequencing. The genome libraries for the isolates were prepared using the Nextera XT Library Prep kit (Ilumina), following the manufacturer’s instructions, and sequenced on an Illumina NextSeq 500 platform with 150 bp paired-end chemistry. The recommended Illumina dual index barcoding (Ilumina) protocol was employed on the generated libraries. Furthermore, read coverage ranging from 18 to 356-fold, with an average of 155-fold was generated in the NexSeq 500. The human DNA sample, sequencing was performed by means of the MiSeq Reagent Kit V3 (2 × 300 bp) on the MiSeq 2000 (Illumina).

#### Quality Assessment and de Novo Assembly

The FastQC software version 0:10.1 (Andrews et al., 2010) was used to access the quality of the sequenced reads. By applying a read > Q28 criterion and Trimmomatic (Bolger et al., 2014), the ambiguous nucleotide reads were eliminated. Using the SPAdes v1.1.0 pipeline, the paired end trimmed reads of *Brucella* spp. strains were de novo assembled (Bankevich et al., 2012). Kmer sizes 21, 33, 55, 77, 99, and 127 were employed for the assembly, with a minimum contig length of 500 bp. The probable contaminants in each assembled genome were also evaluated using CheckM (Parks et al., 2015). For the comparative analysis, reference genomes were selected based on their high-quality assembly, annotation completeness, and representation of known genetic diversity within the *Brucella* genus. The assembled genomes were assessed using Quast v. 2.3 (Gurevich et al., 2013).

#### Multilocus Sequence Typing Analysis (MLST)

The pubMLST database of *Brucella* spp. on the website (https://pubmlst.org/), [accessed on 22-02-2023] was used to determine the species identity of the sequenced genomes. Identification of species and strains was performed by MLST 2.0 and PubMLST. The assignment of the new sequence types (STs) and subspecies identification were achieved by the Pasteur MLST database curators which were implemented on Pathogenwatch platform (Argimón et al., 2021).

#### Pangenomics and Average nucleotide Identity Analysis

Pangenome analysis was conducted using 69 *B. melitensis* strains retrieved from publicly available genome in GenBank that included the 16 sequenced strains in this study. Selection was on the completeness of the genomes, African genomes, and some of the reference genomes i.e., *B. abortus* S19 and *B. melitensis* Rev1 vaccine strains. The pangenome of the *B. abortus* strains was conducted on 56 isolates, that included the three sequenced genomes in this study (Fig. S1). All the retrieved and sequenced *Brucella* spp. strains in this study were further annotated using Prokka v.1.14.0 (Seemann, 2014) and Prodigal 1.20 implemented on Anvi’o v 7.1 (Eren et al., 2021). Similarity searches between the coding domain sequences (CDSs) of assembled genomes were conducted using pairwise BLASTp and Markov Cluster Algorithm (MCL) (Buchfink et al., 2015). The tRNAScan-SE (Chan & Lowe, 2019) was used to determine the number of RNAs. Clusters were created using paralogs of the genomes and were ordered by the presence/absence of orthologs (Page et al., 2016). Pangenome clusters were defined as follows: Core-genes present in all isolates; soft core-genes present in at least 95% of isolates; shell-genes present between 15 and 95% of isolates; cloud-genes in less than 15% of isolates. Assignment of clusters of orthologous groups (COG) was achieved using DeepNOG v. 1.2.3 (Feldbauer et al., 2020). Pangenomics analysis was carried out using Anvio-7.1 (Eren et al., 2021). Average nucleotide identity analysis was conducted using pyANI implemented in Anvio-7.1 (Eren et al., 2021). Phylogenetic trees of the ANI analysis were visualized using the iTOL v5 (Letunic & Bork, 2021).

#### Single Nucleotide Polymorphism and Phylogenetic Analysis

The core-genome alignment generated from the pangenomics analysis was used to determine the SNP for the placement of the sequenced *Brucella* spp. strains. The SNP-sites 2.5.1 (https://github.com/sanger-pathogens/snp-sites) (Page et al., 2016) was used to filter the single nucleotide polymorphism on the core genome alignment. Gubbins (Croucher et al., 2015) was used to identify and remove recombination within the fill alignment. The phylogenetic analysis of *B. abortus* and *B. melitensis* was conducted and the phylogenetic trees were constructed based on the filtered SNPs from the alignment using the IQTREE software (Trifinopoulos et al., 2016) applying the GTR + G substitution model and maximum likelihood methodology (1000 bootstraps). The generated phylogenetic trees were visualized and annotated using the iTOL v5 (Letunic & Bork, 2021).

## Results

### Molecular Identification of the *Brucella* spp. Isolates

A 214 bp fragment was amplified for *Brucella* isolates using the ITS-PCR test, which is used to screen for *Brucella* spp. The AMOS-PCR was used to differentiate and confirm the isolated *Brucella* spp. (n = 19). Of the three cattle samples from Gauteng abattoirs, two were characterised as *B. abortus* and one as *B. melitensis* (Kolo et al., 2019). *Brucella abortus* was isolated from cattle in Gauteng (Ledwaba et al., 2021). The human (Wojno et al., 2016) and goat *Brucella* DNA samples from the Western Cape were identified as *B. melitensis*. *Brucella melitensis* was isolated from sable samples from the Eastern Cape, Western Cape, North West, Limpopo and Gauteng provinces (Glover et al., 2020).

### Whole Genome Assembly and Statistics Measurements

An average of 2,953,271 trimmed reads per sample were observed from the South African *Brucella* spp. isolates. Summary of the genome features of the sequenced *Brucella* spp. are demonstrated in Table 2. The genome sizes of the *B. abortus* in this study had an average of 3,277,013 bp, whereas the *B. melitensis* genome had an average 2,892,570 bp. The genome sizes of the reference *B. abortus* S19 and *B. melitensis* Rev1 were 3,278,310 bp and 3,305,822 bp, respectively. The genomic features of all assemblies including the size, GC content, number of contigs, and CDS are provided in Table 2. The assembly of the sequenced strains resulted in an average of 107 shortest contigs and 347,462 longest contigs. The genome of the reference *B. abortus* S19 and *B. melitensis* Rev1 reported a GC % of 57 and 57.2, respectively. The average GC % of the sequenced *B. abortus* was 57.2, which is comparable to the reference strain S19. The average GC % of the sequenced *B. melitensis* was 57.3, which is approximately the same to the reference strain *B. melitensis* bv 1 Rev1 that is 57%.

**Table 2:** Summary of the genome features of the sequenced *Brucella* spp. strains (n = 19) in this study

Contigs stats	Ba_1_9	Ba_20_S4	Ba_48_S5	Bm_110	Bm_113	Bm_114	Bm_115	Bm_B04serum	Bm_BS0018	Bm_BS0160	Bm_BS0214	Bm_BS0487	Bm_BS04HY	Bm_BS06SERUM	Bm_BS1SKIN	Bm_BS2271	Bm_BS2770	Bm_BmelS8	Bm_NIS8
Total length (bp)	3,299,522	3,265,093	3,266,424	3,279,751	3,277,726	3,278,928	3,286,236	3,278,928	2,910,103	3,018,010	2,980,364	2,884,379	2,885,153	2,944,751	2,898,938	3,018,379	2,945,143	3,290,765	3,283,405
Num Contigs	171	83	159	39	30	38	44	31	23	25	34	23	28	31	89	28	25	287	36
Num Contigs > 100 kb	11	9	3	13	14	12	13	11	9	10	12	11	11	11	4	11	11	0	12
Num Contigs > 50 kb	16	25	20	18	15	17	16	11	11	10	15	11	11	13	21	14	12	3	14
Num Contigs > 20 kb	19	42	56	21	17	21	19	14	13	12	20	14	14	17	44	17	14	51	16
Num Contigs > 10 kb	21	54	84	22	18	22	21	15	15	13	22	15	15	18	59	18	15	118	17
Num Contigs > 5 kb	22	60	106	24	19	23	23	16	16	14	26	16	16	19	67	19	16	192	18
Num Contigs > 2.5 kb	28	69	129	29	21	27	28	20	17	18	29	19	20	24	77	23	20	234	22
Longest Contig	461,691	294,727	136,863	508,856	462,461	638,705	461,83	9,493	518,787	609,698	510,593	518,509	518,534	518,566	164,427	518,55	518,425	58,801	546,03
Shortest Contig	204	204	211	242	241	229	261	429	530	228	226	420	253	287	420	420	491	203	231
Num Genes (prodigal)	3,267	3,167	3,188	3,15	3,143	3,156	3,163	1,561	2,791	2,9	2,869	2,775	2,778	2,839	2,81	2,906	2,833	3,374	3,153
N50	250,134	81,683	44,735	208,49	243,483	221,414	249,77	251,502	324,584	461,969	217,757	251,496	285,772	251,504	62,321	251,503	285,749	18,129	289,912
N75	138,486	49,938	22,311	116,305	171,273	120,416	142,29	143,115	221,856	221,598	119,001	189,159	189,16	143,115	38,409	143,295	186,792	10,587	143,347
N90	74,792	23,923	11,399	71,441	104,619	84,315	71,394	71,445	102,954	105,544	51,875	114,395	105,559	71,445	19,319	71,525	102,27	6,119	102,89
Ribosomal_RNA_16S	1	1	1	1	1	1	1	1	1	1	1	1	1	1	1	1	1	1	1
Ribosomal_RNA_23S	1	1	1	1	1	1	1	1	1	1	1	1	1	1	1	1	1	1	1
Transfer_RNAs	52	52	51	51	51	48	51	46	43	44	42	46	46	46	44	46	46	52	52
GC%	57,27	57,25	57,25	57,27	57,27	57,27	57,27	57,27	57,38	57,31	57,32	57,25	57,25	57,27	57,21	57,28	57,27	57,24	57,24

NB, Ba denotes as *Brucella abortus* genomes, while Bm, as *Brucella melitensis*

### Pangenomes of *Brucella melitensis* and Average Nucleotide Identity (ANI)

The 19 sequenced genomes in this study were classified as *B. melitensis* (n = 16) which include strains Bmel, BS0018, NIS8, BS0214, BS0160, 110, BS0487, BS04SERUM, 113, 114, 115, BS04HY, BS2271, BS2770, BS06SERUM and BS1SKIN. Three of the strains 119, 48 S5 and 20 S4 were identified as *B. abortus* (Table 1). The *B. abortus* sequenced strains were from Gauteng Province, whereas the *B. melitensis* sequenced strains were from across the five selected provinces in SA (Table 1).

**Fig. 1:**
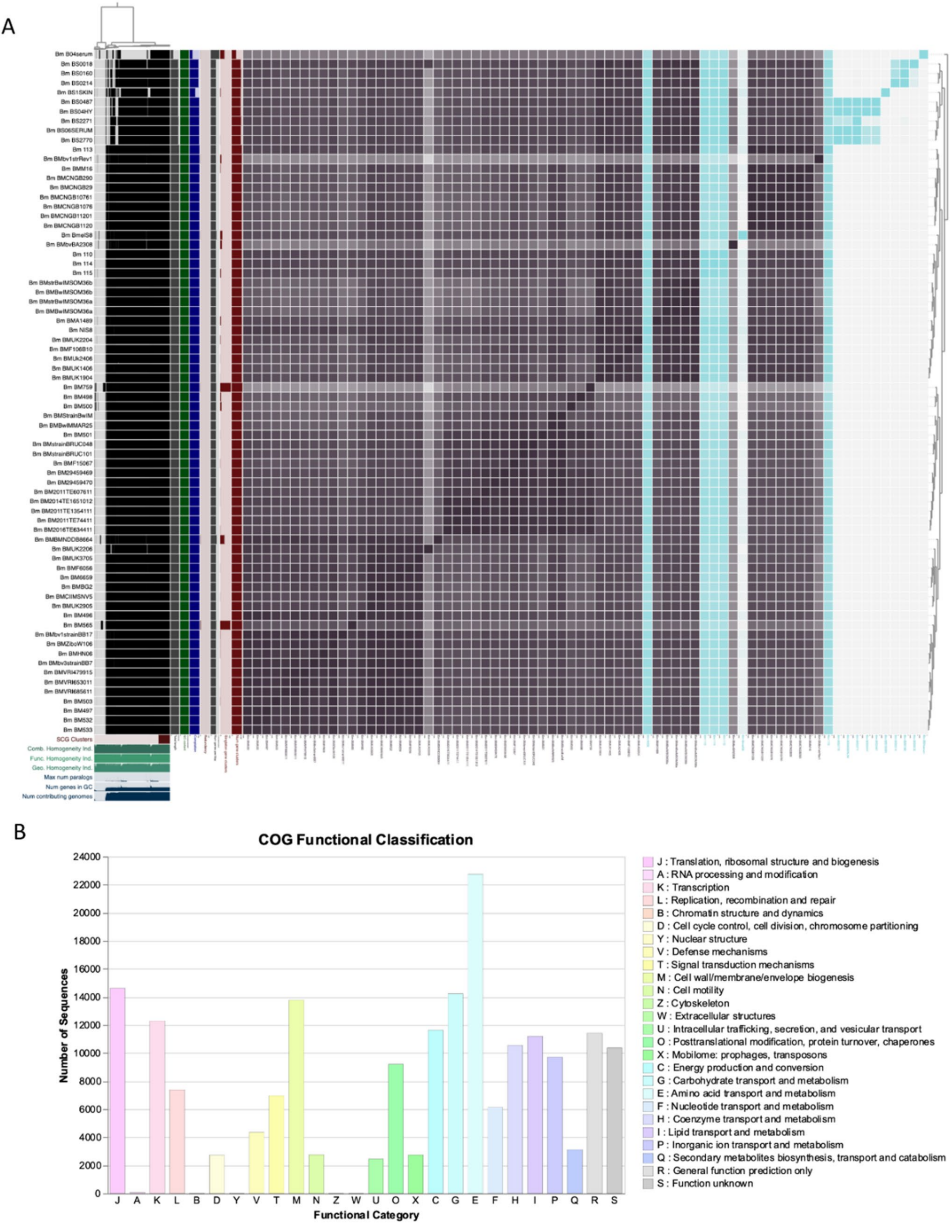
Global pangenome visualisation of the *Brucella melitensis* strains (n = 69) including the sequenced genomes (n = 16) in this study. **A** Phylogeny of the *B. melitensis* indicating the core and accessory genes placing the sequenced genomes highlighted in blue. The number of 3548 gene clusters was observed across 69 genomes. Visualization of pangenome analyses carried by ANVI’O. Central dendrogram clustering of samples is ordered by gene cluster presence/absence. Items order: presence absence (D, Euclidean; L, Ward). **B** COG functional classification of core genome of the analysed-on *B. melitensis*. About 85.24% (190367/223337) sequences classified into COG functional category

The pangenome of the *B. melitensis* genomes (n = 69) was made up of 3,548 total gene clusters that included the sequenced genomes (n = 16) in this study (Fig. 1A). Out of this pangenome, 135 were classified as core-genes. The soft-core genes and shell genes consisted of 1976 and 1330 genes, respectively, meanwhile 5780 were assigned as cloud genes. *B. melitensis* genomes consisted of 85.2% (190367/223337) sequences that were classified into COG functional category (Fig. 1). The COG analysis indicated that 45.27% of the core genes were devoted to metabolism, which were mainly responsible for carbohydrate transport (COG category G), energy production and conversion (COG category C), and amino acid transport and metabolism (COG category E), nucleotide transport (COG category F), coenzyme transport (COG category H), lipid transport (COG category I), and inorganic iron transport (Fig. 1B). All the sequenced South African sable genomes (n = 13) including the cattle (Bmel strain) form their own gene clusters (Fig. 1A). These gene clusters included 10 hypothetical proteins and annotated genes such as Acetyl-coenzyme A synthetase (*acs*), and Acylamidase (*aam*). Among the identified hypothetical proteins assigned with COGs, some i.e., COG5614 and COG3039 are linked with a mobilome prophage HK97 gp10 family protein and transposase IS5 family, respectively. The sequenced BS0018 and NIS8 strains lacked these above-mentioned gene clusters that were isolated from goat and human, respectively. These two genomes including BmelS8 strain are catered by two hypothetical proteins and *cspC* gene that encodes for cold shock-like protein.

The use of average nucleotide identify (ANI) analysis showed that the South African *B. melitensis* sable strains (n = 12) sequenced in this study form their own cluster based on min threshold of > 95%. However, these strains can further be differentiated as strains BS0018 (goat), BS0160 (sable) and BS0214 (sable) grouped together, while BS0487, BSO4HY, BS2271, BS06SERUM and BS2770 form their own sable sub-clade (Fig. 1A). The strain BS1SKIN appeared to be a unique sable strain that is distinctive among the latter mentioned compared clusters of groupings as it was isolated from Limpopo. The three sequenced genomes isolated from sable in Gauteng i.e., strains 1 10, 1 15 and 1 14 form their own cluster that grouped with the BMbvBA2308 strain isolated from Malaysia and the South African cattle BmelS8 strain from Gauteng abattoir. However, BMbvBA2308 strain is highly polymorphic based on SNPs and groups separately from the compared genomes in this cluster. Within this cluster, the sequenced BmelS8 strain that was isolated from the Gauteng abattoir shows a different ANI profile with high number of accessory genes. This strain has 61 hypothetical proteins, and other genes were identified as 3-succinoylsemialdehyde-pyridine dehydrogenase (*ald*), ABC transporter ATP-binding protein Uup, Coenzyme A biosynthesis bifunctional protein CoaBC (*coa*BC), Copper-exporting P-type ATPase (*copA*), Cyclopropane-fatty-acyl-phospholipid synthase (*cfa*), Precorrin-3B C (17)-methyltransferase (*cob*J). Based on ANI which links genes that are shared based on identity, the human case NIS8 strain from Western Cape Province sequenced in this study, clustered with A1489 strain (99.95%) that was isolated from a human sample in Belgium. Sequence types 12 designate both of these human case strains.

### Pangenomes of *Brucella* abortus and Average Nucleotide Identity

**Fig. 2:**
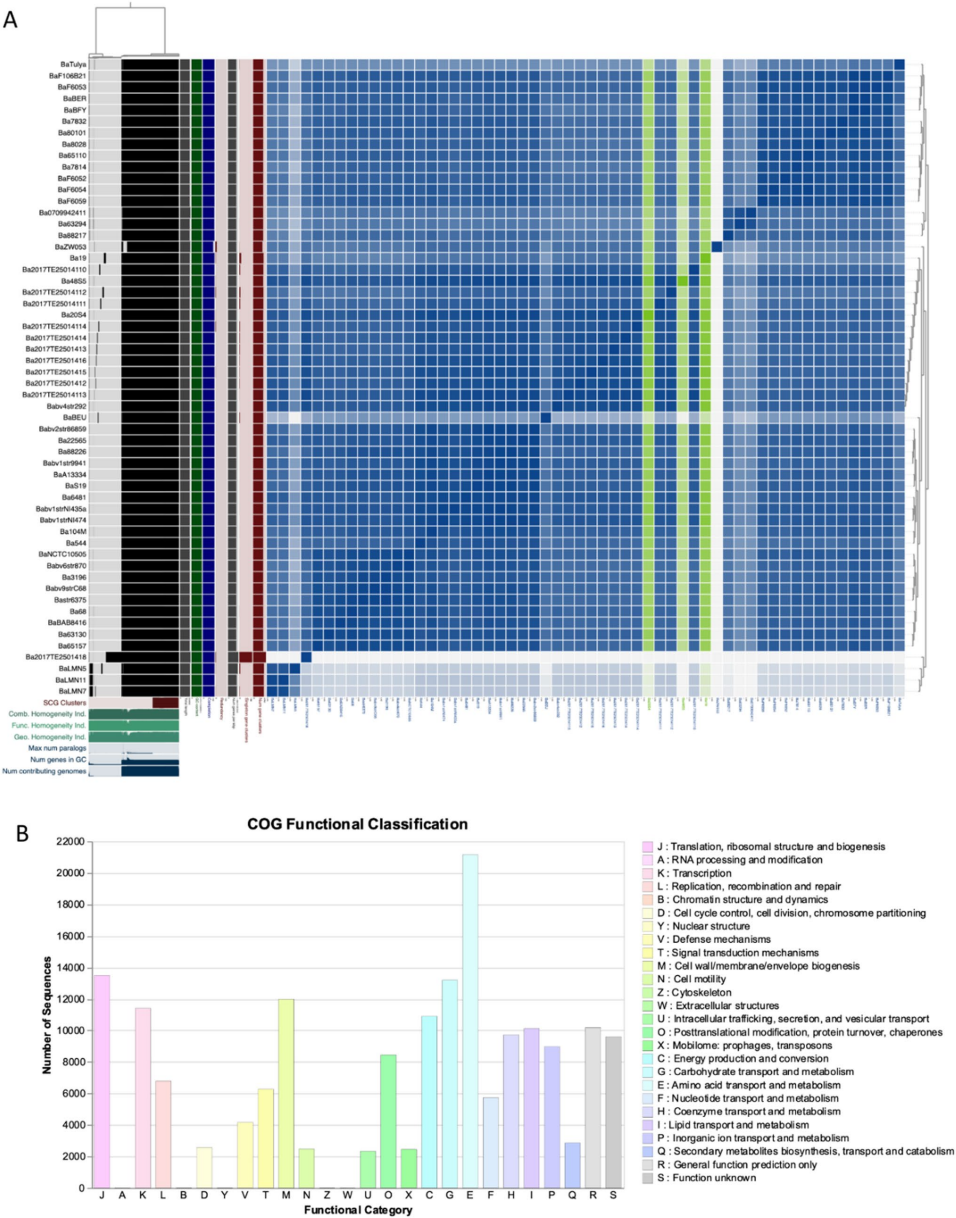
Global pangenome visualisation of the *Brucella abortus* strains (n = 56) including the sequenced genomes (n = 3) in this study. **A** Circular phylogeny of the *B. abortus* indicating the core and accessory genes that constitute of 6,618. The number of gene clusters was observed across 40 genomes. Visualization of pangenome analyses carried by ANVI’O. Dendrogram clustering of samples is ordered by gene cluster presence/absence. Items order: presence absence (D, Euclidean; L, Ward). **B** COG functional classification of core genome of the analysed-on *B. abortus*. About 85.00% (174806/205644) sequences classified into COG functional category

The pangenome of the *B. abortus* strains (n = 56) was made up of 6,618 total genes that placed the sequenced genomes (n = 3) in this study (Fig. 2A). The core genes were defined by 1877, while soft core genes (present in < 99% of the strains) and shell genes (present in < 95% of the strains) consisted of 863 and 564, respectively. The number of cloud genes (present in < 15% of the strains) were assigned as 2935. On the *B. abortus* genomes, 75.43% (174806/205644) sequences were classified into COG functional category (Fig. 2B). The COG analysis indicated that 45.66% of the core genes were devoted to metabolism, which were mainly responsible for carbohydrate transport (COG category G), energy production and conversion (COG category C), and amino acid transport and metabolism (COG category E), nucleotide transport (COG category F), coenzyme transport (COG category H), lipid transport (COG category I), and inorganic iron transport. The three sequenced *B. abortus* strains (48 S5, 19, and 28 S4) clusters with the previously reported South African *B. abortus* strains (n = 10) based on ANI profile.

### Whole Genome Single Nucleotide Polymorphism Analysis (wgSNP) and MLST of *Brucella melitensis*

**Fig. 3:**
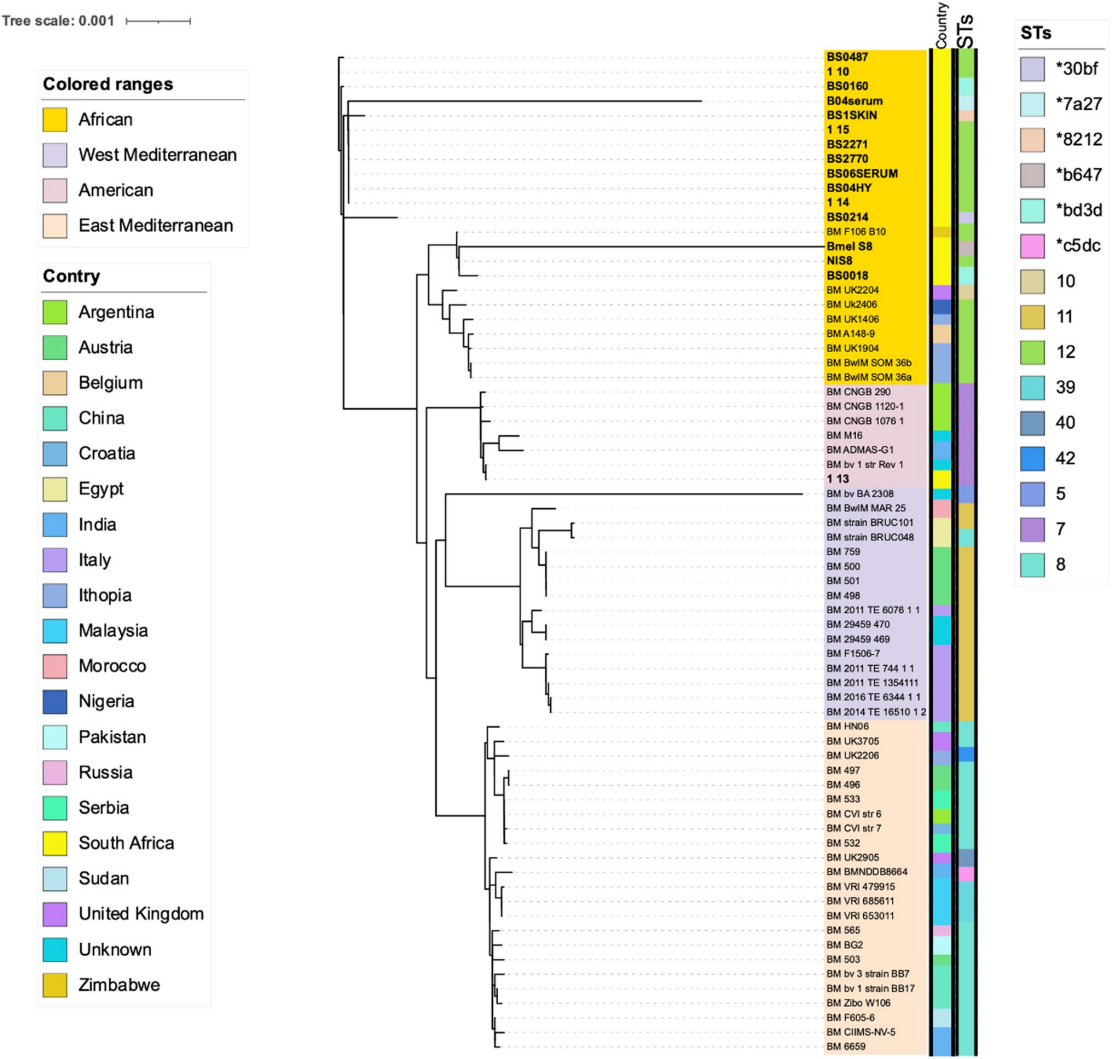
Whole genome single nucleotide polymorphism (wgSNP) phylogenetic tree of *Brucella melitensis* (n = 69) genomes including the sable (n = 13), human (n = 1), cattle (n = 1), and goat (n = 1) sequenced in this study. The wgSNP is based on maximum like hood tree computed using 812 SNPs. Based on wgSNPs, the colour coded keys indicate the isolates’ country of origin as well as sequence types (STs). The scale bar represents number of SNPs. The strains sequenced in this study are highlighted in black

Whole genome SNP tree topology was used to infer the evolution of the sequenced *B. melitensis* (n = 16) with compared global genomes (n = 53) using a total of 845 SNPs that were filtered from the core alignment of the two chromosomes. The sequenced strains in this study cluster in the African lineage. About 234 parsimony informative SNPs defined the phylogenetic tree, clustering them in their respective major clades (Fig. 3). Based on wgSNP phylogenetic analysis of *B. melitensis* genomes indicated that the sequenced NIS8 strain that was isolated from a human in Eastern Cape grouped with the sequenced strains BS0018 (goat) from Eastern Cape and BmelS8 (cattle) from Gauteng abattoir. They form their own African cluster amongst the compared global strains found in this sub-clade that included the Nigerian and Italian strains. Moreover, the human case strain groups closely with the BS0018 strain, isolated from a goat. In contrast, Bmel strain has high polymorphic with high number of non-informative SNPs determined. This strain is distinctive as it was assigned with novel ST as *b647 that was isolated from an abattoir cattle in Gauteng.

All twelve of the *B. melitensis* sable strains from five different provinces group in the African genotype or lineage which forms its’ own subclade. Majority of the African lineage strains are defined by ST 12, while other new STs were determined in the sable strains sequenced in this study. The three strains (1 10, 1 14, and 1 15) isolated from Gauteng sable were assigned as ST 12. Five of the sequenced sable strains of *B. melitensis* (BS2770, BS2271, BS06SERUM, BS04HY, and BS0487) were also assigned with ST 12. Four of the sable strains and a goat strain i.e., B04Serum, BS1SKIN, BS0214, and BS0160/BS0018 were newly assigned as *7a27, *8212, *30bf, and *bd3d, respectively. These strains were obtained from different provinces. The strains BS0214, BS0160, 1 10, and BS0487 form one cluster, while BS04SERUM, 1 14, 1 15, BS04HY, BS2271, BS2770, BS06SERUM and BS1SKIN form their own separate sub clade (Fig. 3, Fig. S1). Strain 1 13 (Rev1 vaccine) that obtained Onderstepoort Biological Products (OBP) was closely related to *B. melitensis* bv 1 Rev1 (vaccine strain) that belongs to the American lineage with ST10 (Fig. 3).

### Whole Genome Single Nucleotide Polymorphism Analysis of *Brucella abortus*

**Fig. 4:**
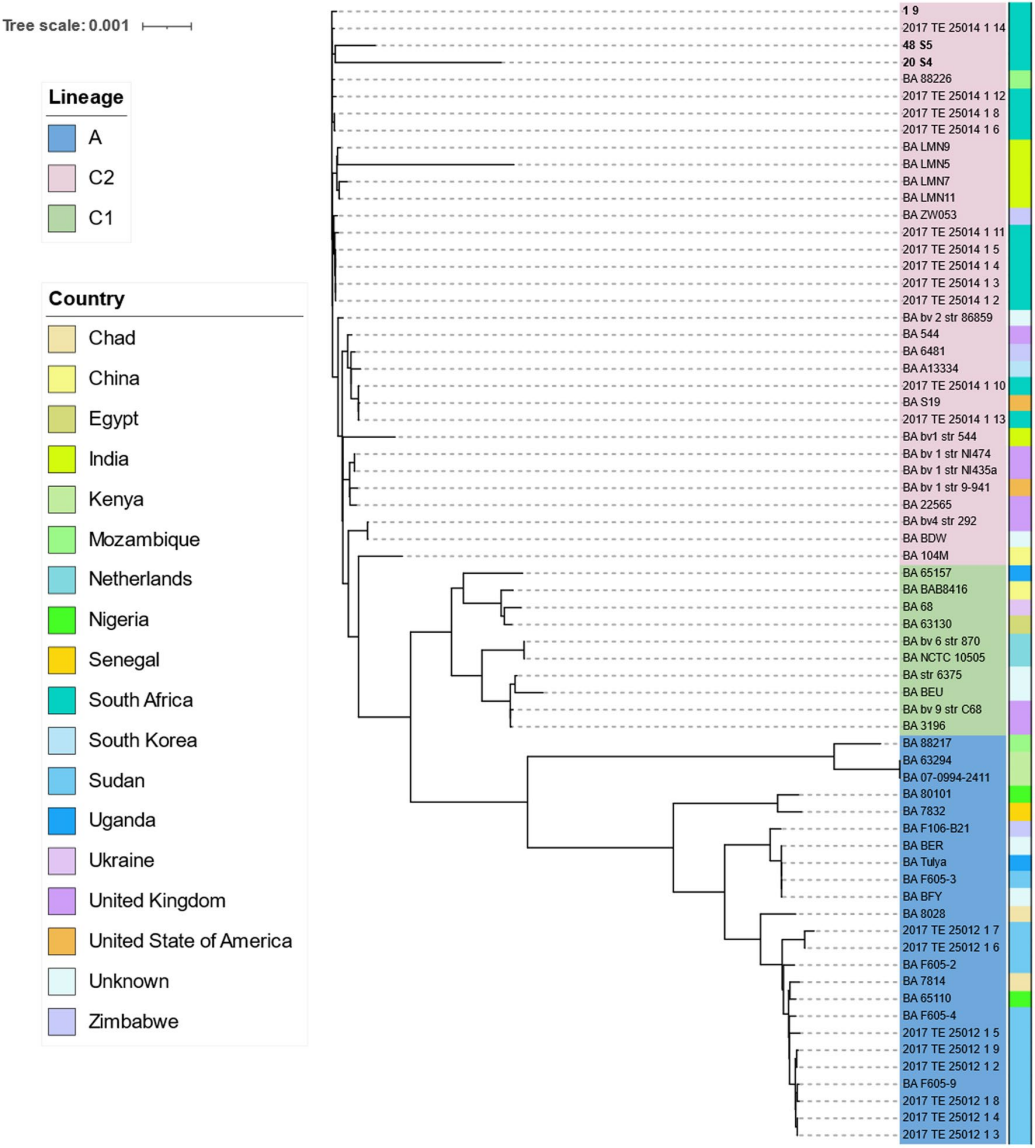
Whole genome single nucleotide polymorphism (wgSNP) phylogenetic tree of *Brucella abortus* (n = 56) genomes including the genomes isolated from cattle sequenced in this study (n = 3). The phylogenetic tree was computed using maximum likelikehood on 1071 SNPs determined accros the compared genomes. Based on wgSNPs, the colour coded keys indicate the isolates’ country of origin and lineage. A. The scale bar represents number of SNPs using branch length

The phylogenetic tree of *B. abortus* (n = 56) was inferred using 1071 core SNPs (Fig. 4). This assigned the lineages into A, C1 and C2. The three *B. abortus* strains (40 S5, 20 S4 and 1.9) sequenced in this study belong to genotype C2 branch with other South African genomes previously reported. Two different genotypes within the C2 branch caters the South African genomes. The Gauteng abattoir *B. abortus* cattle genomes 1.9, 48 S5, and 20 S4 showed to cluster with the South African cattle strain 2017TE25014114 isolated from a farm in Gauteng (Fig. 4). *Brucella abortus* 1.9 and 20 S4 strains were assigned with new STs profiles as *388e and *471f, respectively. Meanwhile, strain 48 S8 was assigned as ST 1. Within this genotype, other SA genomes consisted of 2017TE2S0141 12, 2017TE250141 8, 2017TE2513 1 6, as well as the Mozambique 88/226 strain. Meanwhile strain 1.9 reported from cattle in Gauteng grouped differently amongst the two compared 48 S5 and 20 S4 Gauteng abattoir strains sequenced in this study (Fig. 4B). The second C2 branch genotype caters strains of 2017TE25014 1 11, 2017TE250141 5, 2017TE25014 4, 2017TE250141 3, 2017TE250141 2 and the ZW053 strain of the Zimbabwe. Previously reported related *B. abortus* S19 vaccine strains (2017TE250141 10 and 2017TE250141 13) were also identified in this wgSNP analysis. Among the previously sequenced SA strains (n = 11) that were isolated from Gauteng, the ST 1 is the prevalent sequence type noticeably in the 6 genomes.

## Discussion

As one of the most common zoonotic diseases on the globe, brucellosis necessitates early detection and control to reduce the infection’s incidences and outbreaks. The presence of brucellosis in humans, livestock, and wildlife in SA raises major concerns to public health. Whole genome sequencing was carried on 19 *Brucella* spp. strains to trace the geographical origin of cases of brucellosis circulating in human, cattle, goat, and sable antelope from selected provinces in SA. The *Brucella* spp. reported in this study were identified as *B. melitensis*, which were isolated from human (n = 1) (Wojno et al., 2016), goat (n = 1) (Glover et al., 2020), cattle (n = 1) (Kolo et al., 2019), and sable antelope (n = 12) (Glover et al., 2020) samples obtained from five different South African provinces. The South African *B. melitensis* sable did not cluster with other analysed worldwide genomes but formed a unique cluster in the African clade. This confirms that this infection is a persistent infection circulating in the sable population and not related to livestock as reported by a previous study (Glover et al., 2020), which is further supported by the unique cluster based on ANI and wgSNP analysis. The sequenced *B. abortus* strains are part of the major C2 lineage, which also includes worldwide strains and exhibits two genotypes as previously reported by Ledwaba et al. (2021). Janke et al. (2023) refered to C1 lineage as clade C and C2 lineage as clade D.

The comparative genomics and genome study of *Brucella* spp. involving *B. abortus* strains that are circulating in SA are the subject of very few investigations (Ledwaba et al., 2021). Furthermore, none of the genomes of *B. melitensis* have been examined in SA and contrasted with genomes from around the world. The pangenomic analysis of *B. melitensis* and *B. abortus* revealed a total of 3, 538 and 6,618 gene clusters, respectively. This indicates that *B. abortus* has high number of gene clusters. Both genomes’ species COG analysis indicated that they have > 44% of the core genes devoted to metabolism. It has been observed that these two species have metabolism genes with a percentage greater than 44%, suggesting that the metabolism of energy and amino acids is more crucial to the growth and reproduction processes of *Brucella* species (Yang et al., 2016). The use of pangenomics and ANI analysis showed that the sequenced SA *B. melitensis* genomes from sable and cattle form their own distinct cluster.

Sequenced types were identified among the sequenced genomes in this study from both *B. melitensis* and *B. abortus*. This study demonstrates the relation between the human case strain NIS8 which was the *B. melitensis* strain isolated by Wojno et al. (2016) and goat BS0018 DNA from Western Cape Province. The goat BS0018 isolate was obtained from the Western Cape Provincial Veterinary Laboratory, Stellenbosch. It was isolated from animals testing seropositive during this human outbreak (Wojno et al., 2016). The goat BS0018 strain was collected in Western Cape as to identify the origin of the *B. melitensis* reported in the 27 year old male patient (Wojno et al., 2016). It is evident by the whole genome SNP- based phylogenetic tree that the human strain reported in this study, clustered with the *B. melitensis* BS0018 strain of the Western Cape goat. The isolation reports for *B. melitensis* from human and cattle in SA have not been intensively documented. A small percentage of *B. melitensis* bv 1 (3.3%) and *B. melitensis* bv 3 (18.2%) were reported in SA from samples collected between 2008 and 2018 using laboratory diagnostic data (Matle et al., 2021). An increased isolation of *B. melitensis* was reported in Gauteng (54.5%) followed by 36.4% in the Western Cape Province (Matle et al., 2021). The frequency of *Brucella* reported in Western Cape is contrary to expectations since the province has some of the best regulation and enforcement of animal movement in the country due to foot and mouth control. The human case NIS8 strain from Western Cape sequenced in this study, further clustered with A1489 strain that was isolated from a human sample in Belgium based on ANI and both strains have the sequence types 12. The African genotypes including those in Tanzania are dominant with ST12, while other few of this sequence types have also been reported in India (Bodenham et al., 2020; Karthik et al., 2021). The *B. melitensis* abattoir cattle Bmel S8 strain from Gauteng Province clustered with the human and goat *B. melitensis* strain indicating the circulation of *B. melitensis* in livestock and human in SA. This branch also caters the Gauteng abattoir cattle BmelS8 strain that has a high number of accessory genes. This may be due to the movement of people and animals across provincial borders as this plays a significant role in the infection’s incidence (Sabin et al., 2020).

Studies reporting analyses and trace-back of *Brucella* genomes in SA are very limited. The overall incidence of human brucellosis is unknown in SA (Frean et al., 2018). Only a small number of studies on patients with febrile illnesses and populations at high risk have been carried out in recent years in SA, where there is currently no human brucellosis surveillance program (Frean et al., 2018). Brucellosis patients may exhibit ambiguous signs and symptoms during examination. Patients presenting with symptoms of chronic brucellosis may receive a false diagnosis and inadequate treatment in most developing countries (Wojno et al., 2016). Furthermore, clinicians and laboratory personnel lack awareness and have little expertise diagnosing and managing this pathogen, which causes a delay in diagnosis (Wojno et al., 2016). A recent study conducted by Kolo et al., (2023) reported a brucellosis sero-positivity of 20.4% from abattoir workers in Gauteng Province, SA. A similar study conducted in Gauteng Province amongst workers on cattle farms, reported the seropositivity or exposure to brucellosis using multiple serological screening tests (Govindasamy et al., 2021).

In this study, *B. melitensis* sable samples BS1 skin, B04 Serum and B06 Serum were collected from a farm during an outbreak that occurred in Limpopo by the year 2016. Strain BS2770 grouped among the other sable samples, this was from an outbreak that occurred in 2015 from a farm in North West. Sample BS04HY (2007), BS0487 (2015) and BS0160 (2015) were obtained from the same farm in the Eastern Cape Province during two different outbreaks that occurred in 2007, 2014 and 2015. The findings demonstrated that the outbreaks (in this instance, those from 2007 and 2015) from the same farm persisted there in spite of the quarantine, testing, and slaughter program which was initiated (Glover et al., 2020). Several studies corroborate our findings where the re-emergence of *B. melitensis* infection in wildlife was reported following an eradication period (Garin-Bastuji et al., 2014; Mick et al., 2014). According to Strydom (2016), the brucellosis outbreaks in sables that occurred between 2015 and 2016 in SA was a contributing factor to spillover from livestock to sable. A study conducted by Simpson et al (2021), reported livestock contact as a significant risk factor for brucellosis infection in antelope. This may be attributed by animals (wildlife and livestock) grazing together or sharing water sources during dry seasons (Alexander et al., 2012). This is not supported by the wgSNP analysis in this study as *B. melitensis* sable sequences clustered in separate cluster with *B. melitensis* from any small livestock sequences currently available in this cluster. All the sable genomes cluster in one group based on ANI and represent a new African genotype based on wgSNP analysis. The high number of SNPs found indicate the diverse number of African strains within this population. The sable genomes may be distinguished from the previously published African sequences on GenBank, constituting a unique African sub-clade.

In this study, *B. abortus* isolates obtained from cattle collected in Gauteng abattoirs (20S4, 48S5) were closely related to strains from neighboring countries such as Mozambique and Zimbabwe and further clustered together with the cattle isolate (1.9) from a farm in Gauteng based on wgSNP analysis. This indicates that *B. abortus* is persistant, diverse and circulating within the cattle population in SA. *Brucella abortus* most probably were introduced and/or transmitted to cattle herds through the movement of animals within the country and across borders. It further emphasises that the current bovine brucellosis scheme should be expanded not only to bovine but all host species in SA, including sheep and goats to include compulsory vaccination of not only cattle but also sheep and goats to ensure immunity. The test and slaugtering prescribed by the scheme and expansion to include all bovine and other host species might be more difficult due to capacity and economic constrains in SA.

It is evident that the current bovine brucellosis scheme launched in 1979 has not reduced brucellosis, thus alternative control measures to consider might be mass vaccination of all livestock. In order to execute One Health initiatives against any zoonotic disease, awareness amongst the communities, farmers, health workers and veterinary practitioners is necessary. Lack of awareness regarding animal brucellosis causes ongoing financial losses, the disease's persistence in humans, underdiagnosis and or a delay that raises the risk of complications and treatment failure/relapse in human (Solera et al., 1998). Mass vaccination can effectively reduce outbreaks and spread of brucellosis in an endemic country (Sanz et al., 2010). *Brucella melitensis* has been reported to be effectively controlled by using a mass vaccination with a decreased dose of Rev 1 (Scharp et al., 1999) which can also be considered. Currently, SA is reporting infections of *B. melitensis* in cattle, thus vaccination of livestock could reduce the posibility of future outbreaks and spillover between species.

## Conclusion

Brucellosis remains a controlled zoonotic infection in SA with a reduced proportion of *B. melitensis* reports as compared to *B. abortus*. This study demonstrates a comprehensive analysis of *B. abortus* and *B. melitensis* isolated from human, livestock, and wildlife in SA. We presented a new African branch of *B. melitensis* genotype that is dominant in sable genomes. The presence of *B. melitensis* in human and cattle requires further extensive investigations and revision of control. In conclusion, this study identified genetically diverse *Brucella* spp. among various hosts in SA. The findings of this study will form the foundation for future research on the distribution of the *Brucella* spp. worldwide and its evolutionary background.

The limitation of this study included the difficulties of purifying additional *Brucella* isolates for sequencing, as the livestock samples were collected from apparently healthy animals with no abortion history. According to the literature, it has been reported that the organism is highly concentrated in aborted materials.

## Supplementary Information

Below is the link to the electronic supplementary material.

Supplementary file1 (PDF 155 KB)

## Data Availability

The genomes data is available on NCBI under project PRJNA-1097985.
