# Supplementary material for: Whole Genome Sequence Analysis of Brucella spp. from Human, Livestock, and Wildlife in South Africa
Source: J Microbiol. 2024 Jul 22;62(9):759–73. doi: 10.1007/s12275-024-00155-8 (PMC11436471; doi:10.1007/s12275-024-00155-8)
Supplement: Supplementary file 1 — Supplementary file1 (PDF 155 KB) [file 12275_2024_155_MOESM1_ESM.pdf]

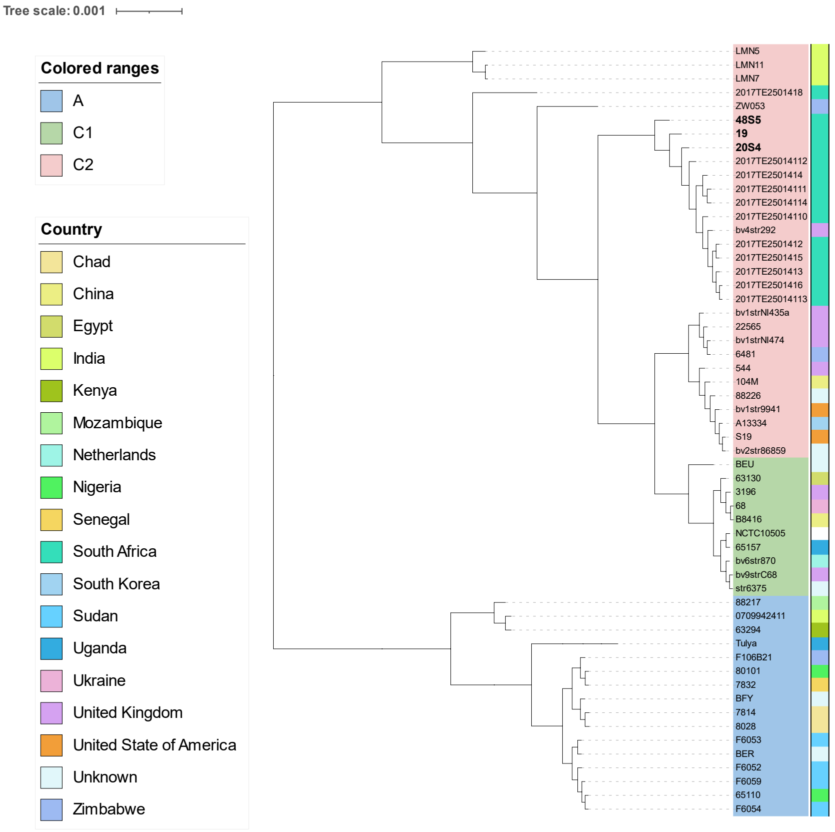

Fig. S1. Phylogram of the *B. abortus* strains based 56 strains clustered according to average nucleotide identity (ANI) showing the clustering.
